# *Prangos ferulacea* (L.) ameliorates behavioral alterations, hippocampal oxidative stress markers, and apoptotic deficits in a rat model of autism induced by valproic acid

**DOI:** 10.1002/brb3.3224

**Published:** 2023-08-18

**Authors:** Maryam Saadat, Abbas Ali Taherian, Mohammad Reza Aldaghi, Payman Raise‐Abdullahi, Hamid Reza Sameni, Abbas Ali Vafaei

**Affiliations:** ^1^ Nervous System Stem Cells Research Center Semnan University of Medical Sciences Semnan Iran; ^2^ Department of Anatomical Sciences, School of Medicine Semnan University of Medical Sciences Semnan Iran; ^3^ Research Center of Physiology Semnan University of Medical Sciences Semnan Iran; ^4^ Department of Physiology, School of Medicine Semnan University of Medical Sciences Semnan Iran

**Keywords:** autism, hippocampus, oxidative stress, *Prangos ferulacea* (L.), valproic acid

## Abstract

**Background:**

Prenatal exposure to valproic acid (VPA) may enhance the risk of autism spectrum disorder (ASD) in children. This study investigated the effect of *Prangos ferulacea* (L.) on behavioral alterations, hippocampal oxidative stress markers, and apoptotic deficits in a rat model of autism induced by valproic acid.

**Methods:**

Pregnant rats received VPA (600 mg/kg, intraperitoneally [i.p.]) or saline on gestational day 12.5 (E 12.5). Starting from the 30th postnatal day (PND 30), the pups were i.p. administered *Prangos ferulacea* (PF, 100 and 200 mg/kg), or the vehicle, daily until PND 58. On PND 30 and 58, various behavioral tasks were used to evaluate pups, including the open field, elevated plus‐maze, hot‐plate, and rotarod test. On PND 65, the animals were euthanized, and their brains were removed for histopathological and biochemical assay.

**Results:**

Prenatal exposure to VPA caused significant behavioral changes in the offspring, reversed by administering an extract of *Prangos ferulacea* (L.). Additionally, prenatal VPA administration resulted in increased levels of malondialdehyde and deficits in antioxidant enzyme activities in the hippocampus, including catalase and glutathione, ameliorated by PF. Likewise, postnatal treatment with PF improved VPA‐induced dysregulation of Bax and Blc2 in the hippocampus and reduced neuronal death in CA1, CA3, and dentate gyrus.

**Conclusion:**

The findings of this study suggest that postnatal administration of PF can prevent VPA‐induced ASD‐like behaviors by exhibiting antiapoptotic and antioxidant properties. Therefore, PF may have the potential as an adjunct in the management of ASD.

## Graphical abstract

Prenatally, valproic acid causes significant behavioral and histopathological disturbances and increases cell death and oxidative stress in the hippocampus. Postnatal treatment of autistic rat pups with *Prangos ferulacea* attenuates the behavioral and histopathological disorders, oxidative stress, and cell death and increases antioxidant enzymes in hippocampus.

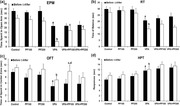

## 1 INTRODUCTION

Autism spectrum disorder (ASD) is a high‐prevalence heterogeneous neurodevelopmental disorder (Baio et al., 2018). In the United States, the prevalence of ASD has been estimated at approximately 1:68 children, and boys are affected 4.5‐fold more than girls (Chiarotti & Venerosi, 2020). ASD has two core symptoms of social communication/interaction and restrictive/repetitive behavior. These core symptoms can be accompanied by other symptoms, such as anxiety, hyperactivity, hypo‐ or hyper‐sensory problems, aggression, epilepsy, attention deficit, mental retardation, intellectual disability, sleep disturbance, and gastrointestinal issues (Mabunga et al., 2015). It has been reported that around 70% of people with autism exhibit cognitive deficits (Pennisi, 2020). Likewise, a link between ASD and morphological characteristics of the hippocampus has been proven (Bauman & Kemper, 1985). For the first time, clinical characteristics of ASD were described during the 1940s (Kanner, 1943). The exact etiology and underlying mechanism of ASD remain poorly understood. It appears that a combination of genetic, epigenetic, environmental, immune, neurochemical, and oxidative stress factors are associated with the symptoms of ASD (Al‐Ayadhi & Elamin, 2013; Eshraghi et al., 2018).

Elevated levels of free radicals, such as reactive oxygen species (ROS), can lead to oxidative stress associated with brain injuries, strokes, and neurodegenerative diseases (Liu et al., 2017). This highlights the critical importance of managing ROS production for normal cellular function.

The cellular defense system includes enzymatic and nonenzymatic antioxidants that play a key role in protecting biological systems from ROS by controlling the production of free radicals and their metabolites. The primary antioxidant enzymes against superoxide radicals are superoxide dismutase, catalase (CAT), and glutathione peroxidase. These enzymes act together in neutralizing the metabolic pathway of ROS (Patlevič et al., 2016 and Sindhi et al., 2013).

Several investigations have suggested a possible link between oxidative stress and the development of ASD (Parker et al., 2017; Rossignol & Frye, 2012). Both oxidative stress and an imbalance between inhibitory and excitatory neurotransmission have been shown to play critical roles in ASD (Toczylowska et al., 2020). The reduced number of neurons via apoptosis considered the leading cause of autism has been attributed to increased levels of ROS in both the brain and plasma (Dong et al., 2018; Russo, 2009). Therefore, we hypothesized that a substance possessing antioxidant and neuroprotective effects could benefit patients with ASD.

Due to a tremendous burden on socioeconomic costs, effective, safe, cheap, and easy‐to‐access interventions are needed. Unfortunately, no effective treatments cure this disorder (Buescher et al., 2014). Risperidone and aripiprazole were approved by the United States Food and Drug Administration to treat irritability in ASD patients without effects on social communication deficits (Kuo & Liu, 2018). There is a growing body of evidence that substances with antioxidant and anti‐inflammatory features, such as resveratrol (Malaguarnera et al., 2020), green tea (Banji et al., 2011), and *Bacopa monnieri* (Sandhya et al., 2012), can also mitigate ASD‐like behavior in the valproic acid (VPA) rat model of autism.

In folk medicine, herbal plants have been used for treating different diseases. Among several medicinal plants, *Prangos ferulacea* (L.) is found in the Mediterranean and Middle‐east regions in large amounts (Sadraei et al., 2013). In Iran, *Prangos ferulacea* (PF), locally known as Jashir, that is traditionally used as a flavoring additive in yogurt (Coruh et al., 2007). PF exhibits various biological activities, including anti‐inflammatory, antioxidant, analgesic, antifungal, antiviral, antidiabetic, cytotoxic, and antispasmodic properties (Coruh et al., 2007; Mavi et al., 2004; Shokoohinia et al., 2014). These biological features are attributed to the high amount of coumarins present in PF (Shokoohinia et al., 2014). Several studies have demonstrated the beneficial effects of coumarins against central nervous system disorders (Skalicka‐Woźniak et al., 2016; Yang et al., 2018). Accordingly, this study aimed to investigate the effects of PF extract on behavioral, histopathological, and molecular brain disorders in a rat model of autism induced by VPA.

## 2 MATERIALS AND METHODS

### 2.1 Animals

The animal study was reviewed and approved by the Ethics Committee of the Semnan University of Medical Sciences (Approval ID: IR.SEMUMS.REC.1399.050), and all procedures were conducted according to the Guide for the Care and Use of Laboratory Animals. During the study, adult Wistar rats and their offspring were kept in standard conditions with free access to water and food, 12 h light/dark cycles, constant humidity (40%–70%), and controlled temperature (25°C).

### 2.2 Animal groups and experimental design

Twenty adult female Wistar rats weighing 180–220 g were randomly selected and placed with 20 mature male rats weighing 200–250 to mate (one female and one male rat in each cage). The day of the presence of vaginal plaque and sperm in the vaginal smear was considered gestational day (GD 0). On ED 12.5 (emberyonic day), the pregnant rats were randomly assigned to the following groups: control group (rats were administered 0.9% saline, 100 μL intraperitoneally [i.p.]); VPA group (rats were administered a single dose of VPA, 600 mg/kg i.p.). On postnatal day 30 (PND 30), 30 young male offspring of mothers in the control group were assigned to the control, PF100 (100 mg/kg, i.p.), and PF200 (200 mg/kg, i.p.) groups. Additionally, 30 young male offspring of mothers exposed to VPA were assigned to the VPA, VPA+PF100, and VPA+PF200 groups (*n* = 10 in each group).

**FIGURE 1:**
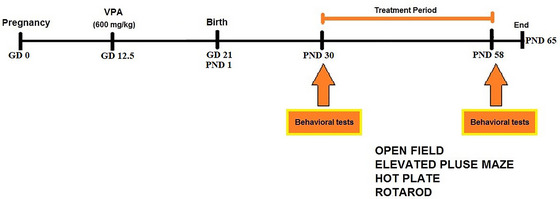
The time line of behavioral training, testing, and drug injections (see Section 2 for more details). GD: gestational day; PND: postnatal day; VPA: valproic acid.

The vehicle (saline) and PF extracts were administered from PND 30 to 58. Behavioral examinations were conducted before the administration of vehicles or PF extracts on PND 30 and 58. On PND 65, rats from each group were sacrificed, and their brains were fixed for histopathological studies. Additionally, the hippocampus was isolated and stored at −80°C for molecular analyses (Figure 1).

### 2.3 Prangos ferulacea (L.)

The aerial parts of the *Prangos ferulacea (L.)* plant were collected in June 2020 from the Semnan area in Semnan Province. The plant was identified by the General Department of Natural Resources of Semnan Province, and a specimen was maintained in the herbarium of the Medicinal Plants Research Center of Semnan University of Medical Sciences, Semnan, Iran, with the code NO. 2020‐100A. The aerial parts of the plant were cut, air‐dried, and ground into a coarse powder.

### 2.4 Preparation of *Prangos ferulacea* (*L*.) extract

The *Prangos ferulacea* (*L*.) extract was prepared following methods described in previous studies (Sadraei et al., 2013). The aerial parts of the plant (500 grams) were extracted with acetone for 2 days (4 × 5 *L*). The resulting extract was concentrated to yield a viscous mass, which was then stored at −20°C for 2 days and dried to form a solid mass.

### 2.5 Behavioral evaluations

#### 2.5.1 Elevated plus‐maze (EPM)

Although anxiety is not a primary clinical manifestation of autism, it is a real challenge for patients with autism (Barendse et al., 2013). Therefore, the effects of PF on anxiety‐like behaviors were evaluated using an elevated plus maze (EPM) task. EPM consists of two opposing enclosed arms and two nonenclosed (open) arms in the shape of a plus sign (50 cm length × 10 cm wide × 40 cm high), elevated approximately 50 cm above the floor. Each rat was placed in the maze's center, facing the open arm, and allowed to explore freely for 5 min. The percent of time spent in the open arm and the number of open‐arm entries were recorded (Moy et al., 2008).

#### 2.5.2 Rotarod test

Since children with autism often exhibit difficulties with motor coordination and skill movements, we aimed to determine the effect of PF on motor coordination using the rotarod test. The animals were placed individually on the apparatus, and the rotating speed was gradually increased by 0.5 cm/second every 5 s. The time taken by each animal to maintain its balance on the rod over 5 min was recorded as the endurance time (Moy et al., 2008).

#### 2.5.3 Open field (OF)

The open field (OF) was a plywood apparatus measuring 70 × 70 × 40 cm^3^, and testing was performed between 9:00 am and 3:00 pm. The animal was placed individually in the OF and allowed to explore it freely for 5 min. Movements were recorded using a digital video camera connected to a computer (Ethovision XT7; Noldus Information Technology BV), and the mean activity during the 5‐min testing session regarding to the present of animal in margine and center of OF were analyzed.

#### 2.5.4 Hot plate

Decreased pain sensitivity is a common feature in children with autism (Hargreaves et al., 1988). Therefore, the pain response to thermal stimuli was evaluated using a new, original Plantar test apparatus for thermal stimulation (Hargreaves Apparatus, Ugo Basile) based on Hargreaves’ method (Corbett et al., 2014). This test was conducted on day 30 postnatal. The withdrawal response latency was recorded to indicate the pain response to thermal stimuli.

### 2.6 Oxidative stress assays

#### 2.6.1 Malondialdehyde (MDA) assay

Malondialdehyde (MDA) is a reactive aldehyde generated during lipid peroxidation, a process that occurs when free radicals and ROS attack and oxidize lipids in cell membranes. MDA is a toxic substance that can cause cell damage and has been implicated in developing various diseases associated with oxidative stress, including cardiovascular disease, neurodegenerative diseases, and cancer. The MDA contents were evaluated in brain tissue samples isolated from rats using a commercially available MDA assay kit (Cat no. S0131; Beyotime Institute of Biotechnology), according to the manufacturer's protocol. Briefly, brain tissue sections were homogenized in 0.15 mL thiobarbituric acid (TBA) diluent, 0.05 mL TBA, and 3 μL antioxidant then heated and boiled for 15 min. Following centrifugation at 1000 × *g* for 10 min at room temperature, the supernatants were collected, and the absorbance of the samples was measured at 532 nm using a microplate reader (Bio‐Rad Laboratories, Inc.). MDA contents were expressed as μmol/mg protein (Zhang et al., 2017).

#### 2.6.2 Glutathione (GSH) assay

Glutathione (GSH) is a powerful antioxidant, playing a crucial role in maintaining cellular redox homeostasis by protecting cells from oxidative damage caused by free radicals and ROS, which can damage lipids, proteins, and DNA. 5,5′‐dithiobis‐(2‐nitrobenzoic acid) was used to detect intracellular GSH levels with a total GSH assay kit (cat no. S0052; Beyotime Institute of Biotechnology), according to the manufacturer's protocol. Briefly, brain tissue sections were homogenized with a glass homogenizer and incubated with the assay solution for 5 min at 25°C. The concentration of GSH was measured spectrophotometrically at 412 nm using a microplate reader (Bio‐Rad Laboratories, Inc.). GSH contents were expressed as μmol/mg protein (Zhang et al., 2017).

#### 2.6.3 Catalase (CAT) assay

CAT is a ubiquitous antioxidant enzyme that plays a critical role in protecting cells from oxidative damage by breaking down hydrogen peroxide (H_2_O_2_) into water and oxygen. Reagents and standards were prepared according to the instructions of the CAT assay kit (ZellBio, GmbH). R1 and R2 reagents were heated to 37°C and 100 μL of R1 reagent along with 10 μL of R2 reagent were added to 10 μL of sample and normal saline, mixed well, and incubated for 1 min. Then 100 μL of reagent R3 and 10 μL of reagent R4 were added and remixed. The absorbance was read using an ELISA reader at a wavelength of 405 nm, and then the amount of CAT was calculated (Abdollahi et al., 2021).

### 2.7 Western blotting

The colonic tissues were homogenized in lysis buffer for 30 min, followed by centrifugation. The supernatant was collected, and the protein concentration (Bax and Bcl‐2) was determined with the BCA method. In addition, the Bax/Bcl‐2 ratio was calculated in each group. The supernatant containing 50 μg of protein was then boiled for 5 min and then subjected to sodium dodecyl sulfate polyacrylamide gel electrophoresis for protein separation. Then, the proteins were transferred onto nitrocellulose membranes that were then treated with 5% nonfat milk in TBST containing 10 mmol/L Tris–HCl (pH 7.5), 150 mmol/L NaCl, and 0.05% Tween‐20 at room temperature to block nonspecific reactivity for 1 h. The following primary antibodies were used: occludin (1:1000, ABclonal, no: A12621), claudin‐1(1:1000, ABclonal, no: A2196), and anti‐GAPDH (1:1000, Boster Biotechnology, BM1985) (Huang et al., 2017).

### 2.8 Nissl staining

After the perfusion of the animal, the brain was fixed in the secondary fixative solution for 12–24 h. Then, tissues were washed with tap water and dehydrated. After tissue processing using a tissue passage machine, the specimens were embedded in paraffin, and blocks were sectioned at 5 μm using a rotary microtome. The tissue sections were collected on glass slides and stained with Cresyl violet. Then, the optical microscope (Olympus) examined sections with ×400 magnification. The pyramidal neurons with clear and distinct nuclei were considered healthy cells. Eight photomicrographs were prepared from each sample, and three photomicrographs with a minimum distance of 40 μm were randomly selected, and the pyramidal cells of CA1, CA3, and DG of the hippocampus were counted by Image Tools 2 software (Navaie et al., 2018).

### 2.9 Statistical analysis

Statistical analysis was performed using SPSS version 22.0 (Statistical Product and Service Solutions Inc.). The data are expressed as mean ± standard error of the mean. Three‐way analysis of variance (ANOVA), followed by Tukey's post hoc test, was used to compare groups. A value of *p* < .05 was considered statistically significant.

## 3 RESULTS

### 3.1 Effects of *Prangos ferulacea* (*L*.) on behavioral tests in VPA‐induced model of autism

#### 3.1.1 EPM results

**FIGURE 2:**
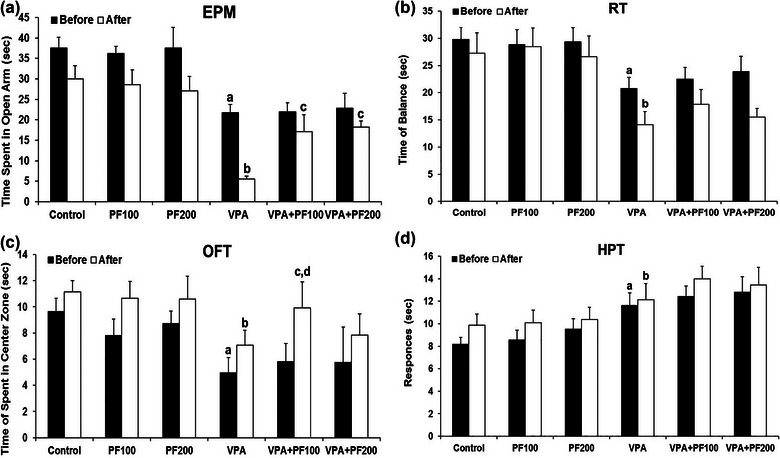
The effects of *Prangos ferulacea*e (L.) on (a) anxiety‐like behaviors in the elevated plus maze (EPM), (b) motor balance in the rotarod (RT), (c) anxiety‐like behavior in the open field (OFT), and (d) pain responses in the hot plate (HPT) in offspring rats prenatally exposed to valproic acid (VPA) before and after treatment. Data are expressed as mean ± standard error of the mean (SEM): (a) *p* < .01 versus the control group, (b) *p* < .05 versus the PF100 group, (c) *p* < .01 versus the PF200 group, (d) *p* < .05 versus the VPA group. Control: healthy rats, PF100 and PF200: healthy rats received PF100 or PF200 (mg/kg), VPA: prenatally VPA‐exposed rats that received the vehicle, VPA+PF100 and VPA+PF200: prenatally VPA‐exposed rats that received PF100 or PF200 (mg/kg).

A three‐way ANOVA [with three variables: (1) period of investigation (pre‐ or posttreatment with PF); (2) maternal VPA or saline treatment groups; and (3) postnatal PF treatment groups (treatment with PF or vehicle)] on time spent in the open arm showed significant effects of the period (*F*_1, 84_ = 29.23, *p* < .0001), and VPA treatment (*F*_1, 80_ = 96.97, *p* < .0001), no significant effect of PF treatment (*F*_2, 84_ = 1.55, *p* = .2) and no significant interaction between period and VPA treatment (*F*_1_, _84_ = .02, *p* = .8), period and PF treatments (*F*_2, 84_ = .93, *p* = .39), a significant interaction between VPA and PF treatments (*F*_2, 84_ = 3.41, *p* = .03) and no significant interaction between period, VPA, and PF treatments (*F*_2, 84_ = 1.65, *p* = .19). Between‐group comparisons demonstrated that the time spent in the open arm in the VPA‐offspring‐vehicle group was significantly shorter than in the saline‐offspring group (*p* < .01). These findings indicated that maternal VPA caused anxiety‐like behavior in offspring. Also, findings indicated that administering PF modulated the anxiety‐like behavior in VPA rats offspring (Figure 2a).

#### 3.1.2 Rotarod test results

A three‐way ANOVA on the rotarod motor and balance test results showed the significant effects of the period (*F*_1, 84_ = 6.92, *p* = .01), and VPA treatment (*F*_1, 80_ = 33.59, *p* < .0001), no significant effect of PF treatment (*F*_2, 84_ = .27, *p* = .75), no significant interactions between period and VPA treatment (*F*_1, 84_ = 2.71, *p* = .14), and period and PF treatments (*F*_2, 84_ = .32, *p* = .72), a significant interaction between VPA and PF treatments (*F*_2, 84_ = .31, *p* = .72), and no significant interaction among period, VPA, and PF treatments (*F*_2, 84_ = .02, *p* = .97). Between‐group comparisons demonstrated that the duration of maintaining balance in rotarod was significantly shorter in the VPA‐offspring compared to the saline‐offspring group (*p* < .01). These findings indicated that the maternal VPA treatment had caused instability in maintaining balance in offspring. Also, findings indicated that administering PF modulated the maintaining balance in VPA rats offspring (Figure 2b).

#### 3.1.3 OF results

A three‐way ANOVA on OF results showed the significant effects of the period (*F*_1, 84_ = 9.21, *p* = .003) and VPA treatment (*F*_1, 80_ = 12.80, *p* = .0006), no significant effect of PF treatment (*F*_2, 84_ = .07, *p* = .92), no significant interactions between period and VPA treatment (*F*_1, 84_ = .19, *p* = .66), period and PF treatments (*F*_2, 84_ = .45, *p* = .63), VPA and PF treatments (*F*_2, 84_ = 1.16, *p* = .31), and period, VPA, and PF treatments (*F*_2, 84_ = .03, *p* = .96). Between‐group comparisons demonstrated that the time spent in the center of OF in the VPA‐offspring vehicle was significantly shorter than the saline‐offspring group (*p* < .01). These findings indicated that the maternal VPA treatment caused anxiety‐like behavior in offspring. Also, findings indicated that administering PF modulated the anxiety‐like behavior in VPA rats offspring (Figure 2c).

#### 3.1.4 Hot‐plate results

A three‐way ANOVA on thermal pain threshold showed significant effects of the period (*F*_1, 84_ = 3.07, *p* = .08), VPA treatment (*F*_1, 80_ = 24.14, *p* < .0001), no significant effect of PF treatment (*F*_2, 84_ = 1.02, *p* = .36), no significant interactions between period and VPA treatment (*F*_1, 84_ = .13, *p* = .71), period and PF treatments (*F*_2, 84_ = .13, *p* = .87), VPA and PF treatments (*F*_2, 84_ = .21, *p* = .81), and period, VPA, and PF treatments (*F*_2, 84_ = .08, *p* = .91). Between‐group comparisons demonstrated that the pain threshold in the VPA‐offspring‐vehicle group was significantly longer than in the saline‐offspring group (*p* < .01). This finding indicated that maternal VPA treatment increased the pain threshold. Also, findings indicated that administering PF reduced the thermal pain threshold in VPA rats offspring (Figure 2d).

### 3.2 The effects of PF extract on the hippocampal levels of oxidative stress markers (MDA, GSH, and CAT)

A two‐way ANOVA (VPA and PF treatment groups) on MDA levels showed a significant effect of VPA treatment (*F*_1, 33_ = 32.60, *p* < .0001) but no significant effect of PF treatment (*F*_2, 33_ = 1.85, *p* < .17) and no significant interaction between VPA and PF treatment groups (*F*_2, 33_ = .49, *p* = .61).

A two‐way ANOVA on GSH levels showed significant effects of VPA treatment (*F*_1, 33_ = 24.57, *p* < .0001) and PF treatment (*F*_2, 33_ = 7.60, *p* < .001) but no significant interaction between VPA and PF treatment groups (*F*_2, 33_ = .001, *p* = .99).

**FIGURE 3:**
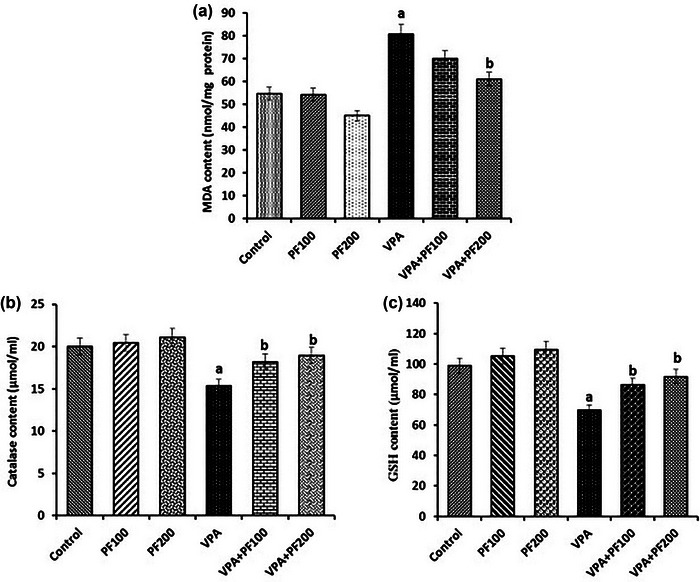
The effects of *Prangos ferulacea*e (L.) on hippocampal levels of (a) malondialdehyde (MDA), (b) catalase (CAT), and (c) glutathione (GSH) in a valproic acid (VPA)‐induced model of autism. Data are expressed as mean ± standard error of the mean (SEM): (a) *p* < .01 versus the control group, (b) *p* < .05 versus the VPA group, (c) *p* < .05 versus the VPA+PF100 group. Control: healthy rats, PF100 and PF200: Healthy rats received PF100 or PF200 (mg/kg), VPA: prenatally VPA‐exposed rats that received the vehicle, VPA+PF100 and VPA+PF200: prenatally VPA‐exposed rats that received PF100 or PF200 (mg/kg).

A two‐way ANOVA on CAT levels showed a significant effect of VPA treatment (*F*_1, 33_ = 8.51, *p* < .006) but no significant effect of PF treatment (*F*_2, 33_ = 1.07, *p* < .35) and no significant interaction between VPA and PF treatment groups (*F*_2, 33_ = .22, *p* = .8). Between‐group comparisons demonstrated that the amount of MDA in the hippocampus of the VPA‐offspring vehicle group was significantly higher, but GSH and CAT levels were significantly lower than the saline‐offspring group (*p* < .01). Also, findings indicated that administering PF modulated the oxidative stress markers in VPA rats offspring (Figure 3a–c).

### 3.3 The effects of PF extract on protein levels of hippocampal Bax, Bcl‐2, and Bax/Bcl‐2 ratio

Western blot analysis was used to investigate the hippocampal mean protein levels of Bax and Bcl‐2 in a VPA‐induced model of autism.

A two‐way ANOVA (VPA and PF treatment groups) on the Bax levels showed a significant effect of VPA treatment (*F*_1, 12_ = 120.27, *p* < .0001) but no significant effect of PF treatment (*F*_2, 12_ = .76, *p* < .48) and no significant interaction between VPA and PF treatment groups (*F*_2, 12_ = .14, *p* = .86).

A two‐way ANOVA (VPA and PF treatment groups) on the Bcl‐2 levels showed a significant effect of VPA treatment (*F*_1, 12_ = 50.34, *p* < .0001) but no significant effect of PF treatment (*F*_2, 12_ = 0.2, *p* < .81) and no significant interaction between VPA and PF treatment groups (*F*_2, 12_ = .19, *p* = .82).

A two‐way ANOVA (VPA and PF treatment groups) on Bax/Bcl‐2 ratio showed a significant effect of VPA treatment (*F*_1, 12_ = 20.47, *p* = .0007) but no significant effect of PF treatment (*F*_2, 12_ = .39, *p* < .68) and no significant interaction between VPA and PF treatment groups (*F*_2, 12_ = .30, *p* = .73).

**FIGURE 4:**
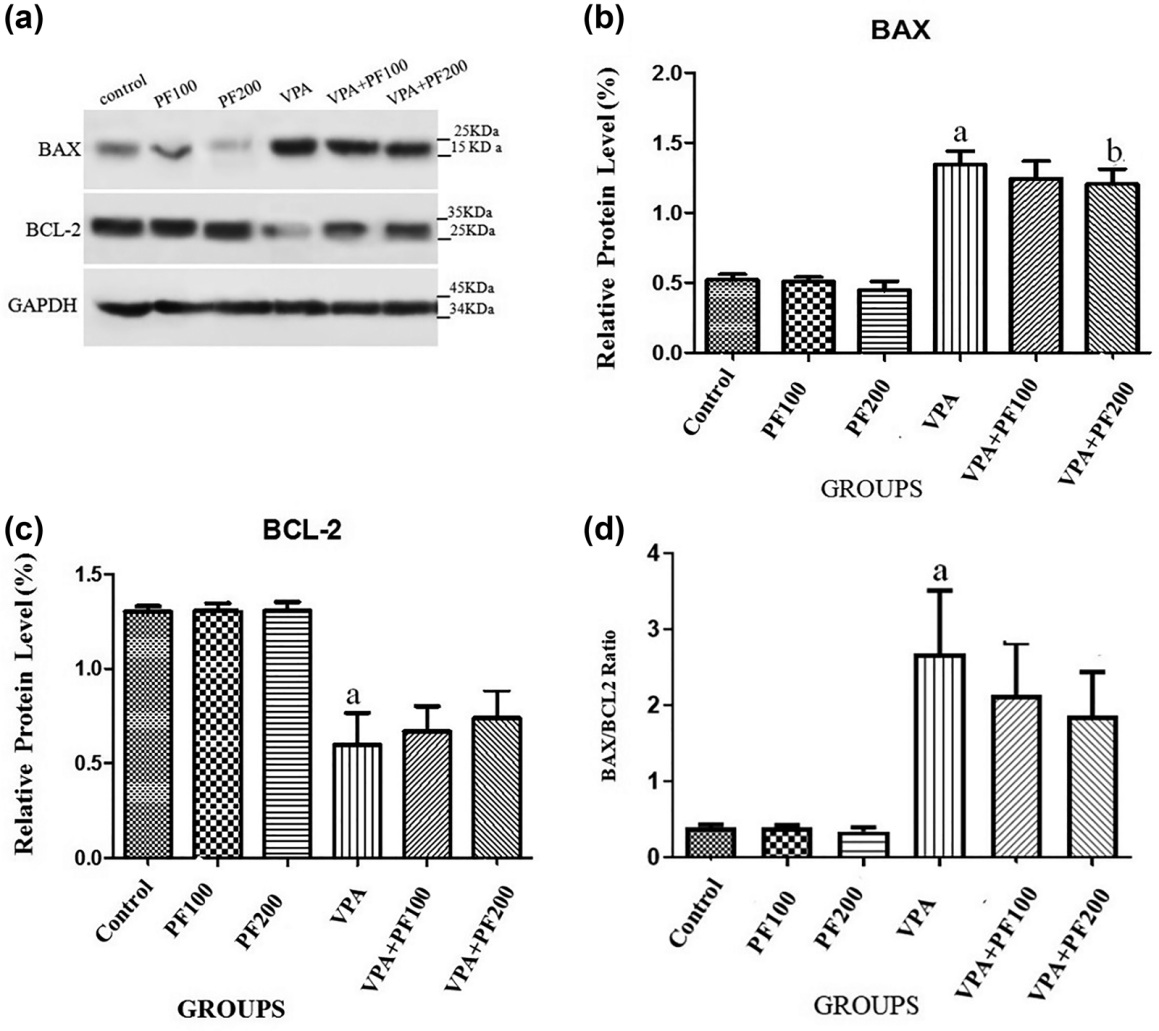
The effects of *Prangos ferulacea*e (L.) on the hippocampal protein levels of (a and b) Bax, (a and c) Bcl‐2, and (d) the Bax/Bcl‐2 ratio in a valproic acid (VPA)‐induced model of autism were analyzed through immunoblotting. Data are expressed as mean ± standard error of the mean (SEM): (a) *p* < .01 versus the control group, (b) *p* < .05 versus the PF100 group, (c) *p* < .01 versus the PF200 group. Control: healthy rats, PF100 and PF200: Healthy rats received PF100 or PF200 (mg/kg), VPA: prenatally VPA‐exposed rats that received the vehicle, VPA+PF100 and VPA+PF200: prenatally VPA‐exposed rats that received PF100 or PF200 (mg/kg).

Between‐group comparisons revealed that the hippocampal levels of Bax were significantly higher, whereas Bcl‐2 levels were lower, and the Bax/Bcl‐2 ratio was higher in the VPA‐offspring vehicle group compared to the saline‐offspring group (*p* < .01). Also, findings indicated that administering PF no significant effect on the dysregulation of Bax and Blc2 levels in VPA rats offspring (Figure 4a–d).

### 3.4 The effects of PF on the percentage of neuronal death in the hippocampal CA1, CA3, and DG subregions

Nissl staining was performed to evaluate the effects of PF on the neuronal death of the hippocampal CA1, CA3, and DG subregions in a VPA‐induced model of autism.

A two‐way ANOVA (VPA and PF treatment groups) on the percentage of CA1 neuronal death showed significant effects of VPA treatment (*F*_1, 12_ = 519.86, *p* < .0001) and PF treatment (*F*_2, 12_ = 92.37, *p* < .0001) and significant interaction between VPA and PF treatment groups (*F*_2, 12_ = 122.03, *p* = 0.0001).

A two‐way ANOVA on the percentage of CA3 neuronal death showed significant effects of VPA treatment (*F*_1, 12_ = 76.94, *p* < .0001) and PF treatment (*F*_2, 12_ = 19.59, *p* = .0002) and significant interaction between VPA and PF treatment groups (*F*_2, 12_ = 22.82, *p* < .0001).

A two‐way ANOVA on the percentage of DG neuronal death showed significant effects of VPA treatment (*F*_1, 12_ = 126.76, *p* < .0001) and PF treatment (*F*_2, 12_ = 29.64, *p* < .0001) and significant interaction between VPA and PF treatment groups (*F*_2, 12_ = 40.37, *p* = .0001). Between‐group comparisons demonstrated that the percentage of neuronal death in the hippocampal CA1, CA3, and DG subregions of the VPA‐offspring vehicle group was significantly higher compared to the saline‐offspring group (*p* < .01). Also, findings indicated that administering PF modulated the number of neuronal death in the hippocampal CA1, CA3, and DG subregions in VPA rats offspring (Figure 5a–c).

FIGURE 5The effects of *Prangos ferulacea*e (L.) on the percentage of neuronal death in the hippocampal CA1, CA3, and DG subregions in a valproic acid (VPA)‐induced model of autism. Data are expressed as mean ± standard error of the mean (SEM): (a) *p* < .001 versus the control group, (b) *p* < .01 versus the VPA group, (c) *p* < .05 versus the VPA+PF100 group. Control: healthy rats, PF100 and PF200: Healthy rats received PF100 or PF200 (mg/kg), VPA: prenatally VPA‐exposed rats that received the vehicle, VPA+PF100 and VPA+PF200: prenatally VPA‐exposed rats that received PF100 or PF200 (mg/kg), Nissl staining, (the scale bars are as follows: row 1 = 200 μm, row 2 = 100 μm, and row 3 = 20 μm, respectively).

**Figure:**
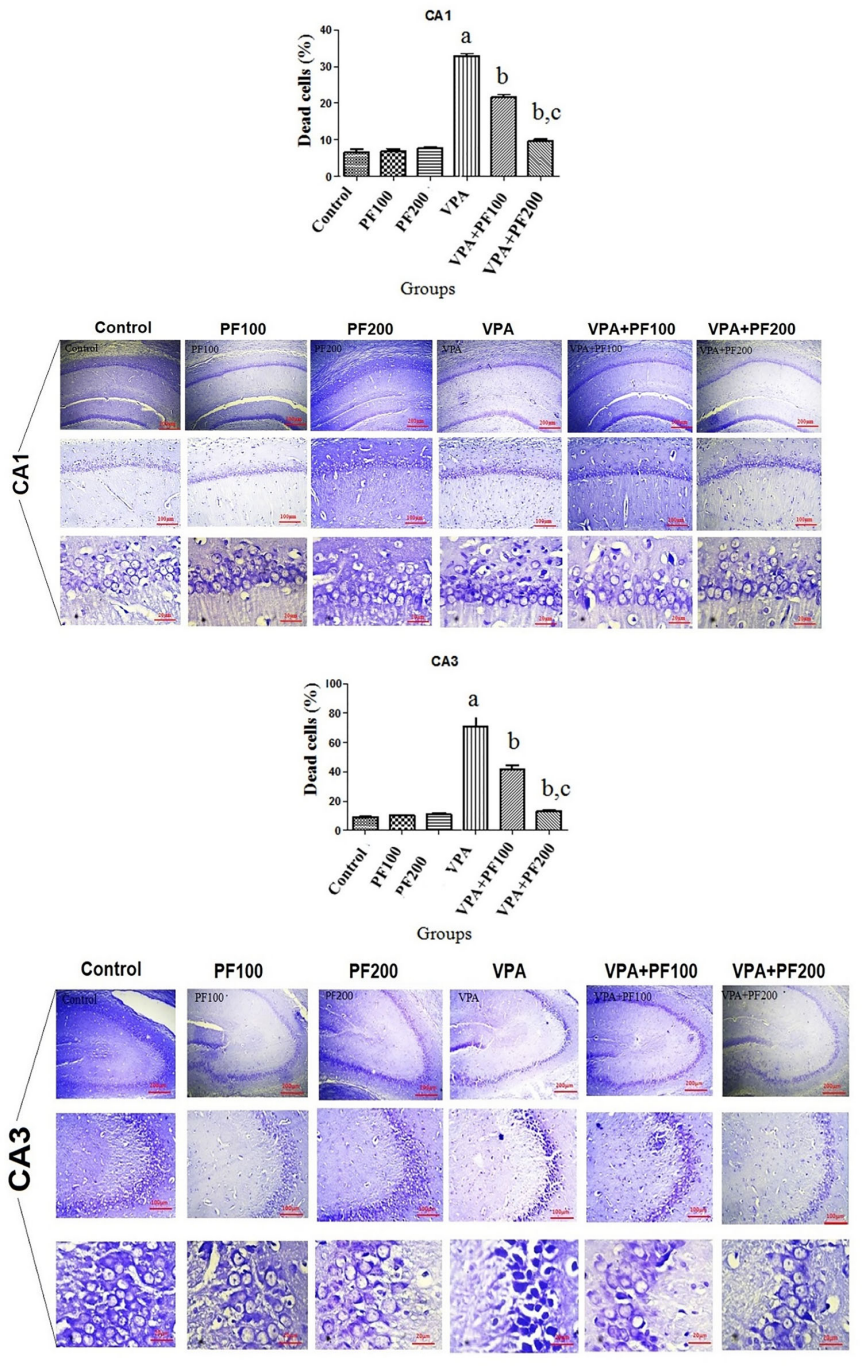

**Figure:**
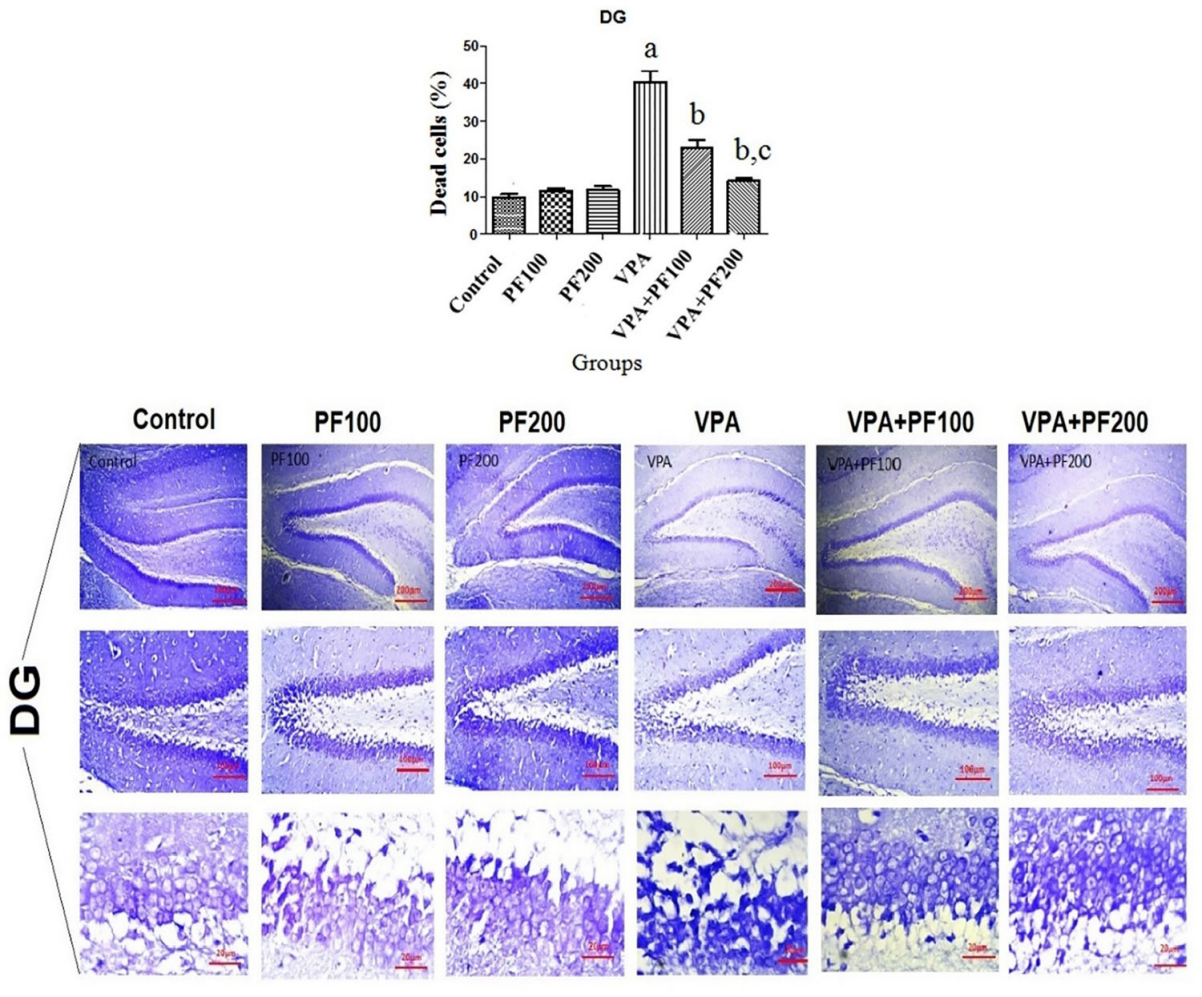

## 4 DISCUSSION

This study evaluated the effects of PF on behavioral, histopathological, and molecular alterations in VPA‐induced autistic animals. Pups from VPA‐exposed mothers received PF (100 and 200 mg/kg) for 4 weeks, beginning 30 days after birth. Results showed that injecting mothers with 600 mg/kg VPA led to autistic‐like behavioral changes in their pups, including increased anxiety and depression, decreased motor coordination, and thermal hyperalgesia. Furthermore, an increased number of neural deaths were observed in the hippocampal CA1, CA3, and DG regions, along with dysregulated Bax/Bcl‐2 proteins and increased levels of ROS in different hippocampal regions. Increased levels of hippocampal MDA, an indicator of lipid peroxidation, and decreased levels of antioxidants GSH and CAT were also confirmed in autistic rats.

ASD is a heterogeneous group of neurodevelopmental disorders associated with severe communication deficits and repetitive/stereotyped behaviors, accompanied by sensory deficits and other comorbidities (Graciarena et al., 2019). According to the literature, the prenatal administration of VAP is a standard model of autism to study the underlying mechanisms of disease and evaluate the effects of several therapeutics. A wide range of behavioral characteristics can be found in the VPA rats, such as deficit in social interaction and repetitive/stereotypic‐like activity correlated with ASD (Bambini‐Junior et al., 2011). Exposure of rat fetuses to VPA resulted in diminished sensitivity to pain and enhanced sensitivity to non‐painful stimuli (Mabunga et al., 2015). Clinically, ASD is also accompanied by comorbidities, including anxiety and cognitive disorders (Hou et al., 2018). These deficits correlate with abnormalities in many brain regions in ASD cases. Neural death in pyramidal neurons of CA1 and CA3 has been confirmed in several studies (Gao et al., 2016; Raymond et al., 1995).

Similarly, the reduced thickness of the hippocampus was confirmed in VPA‐induced animals (Sosa‐Díaz et al., 2014). In addition, prenatal exposure to VPA has been associated with hyperexcitability of hippocampal CA1 pyramidal neurons, which can lead to ASD‐like behaviors (Hajisoltani et al., 2019). ASD is characterized by an imbalance in levels of oxidative biomarkers and lower levels of antioxidant biomarkers in the brain and peripheral tissues (Smaga et al., 2015). Reportedly, prenatal VPA administration increased MDA and nitrite levels and reduced antioxidant enzyme activity in the hippocampus and prefrontal cortex (Ishola et al., 2020). Previous studies have shown that ROS is critical in neurodegeneration during development (Tung & Winn, 2011). Therefore, we suggest that the deficits in learning, memory, and social behavior observed in VPA‐induced pups in this study may be due to enhanced ROS, which could lead to the degeneration of hippocampal pyramidal neurons. ROS results in lipid peroxidation, enzyme inactivation, the break of DNA strands, covalent binding to protein, nucleic acid, and other damaging effects (Yui et al., 2016). In addition, GSH not only serves as an antioxidant but also plays a crucial role in neural survival during the early critical stage (Yui et al., 2016). It has been shown that pyramidal neurons are the first cells affected by ROS (Perry et al., 2004). Bcl‐2 family proteins (e.g., Bax and Bcl‐2) are regulatory proteins responsible for controlling programmed cell death in the brain (Krajewska et al., 2002). A possible association has been revealed among neural cell death, apoptotic mechanisms, and autism, which are involved in the pathophysiology of autism (Wei et al., 2014). On the other hand, increasing ROS biomarkers in the brain and peripheral plasma is related to the reduced number of brain cells and autistic symptoms (Mahmood et al., 2018). Notably, agents with the antioxidant and antiapoptotic characteristics may alleviate ASD symptoms by regulating oxidative stress and ROS production and reducing apoptosis in the neurons of CA1, CA3, and DG regions.

The therapeutic effects of naturally occurring components against autistic behaviors were investigated in several studies. Reportedly, leptin and camel milk administration alone or in combination have ameliorated the expression of ROS and inflammatory biomarkers, including caspase‐3, Bax, and tumor necrosis factor‐α (Hamzawy et al., 2018). Furthermore, using compounds present in plants with antiinflammatory and antioxidant activities in the brain is beneficial for treating ASD‐like abnormalities (Pangrazzi et al., 2020). Several studies have confirmed the therapeutic effects of green tea in ameliorating autistic behaviors through regulating pro‐inflammatory molecules such as TNF and C‐reactive protein (Banji et al., 2011; Lardner, 2014). Supplementation with curcumin (the active component of turmeric) increased concentrations of antioxidant enzymes in the VPA‐induced rats model of autism (Al‐Askar et al., 2017).

In this study, we evaluated the antioxidant and antiapoptotic effects of PF at two doses of 100 and 200 mg/kg on autistic‐like behaviors induced by prenatal exposure to VPA. The results showed that both doses of PF successfully improved anxiety in ASD animals by reducing the number of dead neurons in the hippocampal CA1, CA3, and DG regions. Furthermore, decreased lipid peroxidation (as evidenced by reduced MDA levels) and enhanced antioxidant activities (as evidenced by elevated CAT and GSH levels) were observed in the hippocampal region of ASD pups. Additionally, the results demonstrated that PF has antiapoptotic properties by regulating Bax and Bcl‐2 gene expression. Therefore, PF exerted its beneficial effects via its antioxidant and antiapoptotic properties.

The previous studies have demonstrated the antioxidant effect of PF extract in inhibiting lipid peroxidation and scavenging free radicals. The antioxidant activities of PF were found to be higher than vitamin E due to its effects on GSH‐*S*‐transferase activity (Coruh et al., 2007). The main components of PF extract have been identified as monoterpenes, especially alpha‐ and beta‐pinene. The presence of active components, including monoterpenes, sesquiterpenes, coumarins, flavonoids, tannins, saponins, alkaloids, and terpenoids, leads to antioxidant, antidiabetic, antimicrobial, antiviral, anti‐analgesic, and antispastic properties (Kafash‐Farkhad et al., 2013). The phenolic compounds of this plant confirm its high antioxidant properties (Coruh et al., 2007; Razavi et al., 2009). It has been reported that aqueous and methanolic extracts of PF reduced pain in formalin‐induced pain in mice. These effects were related to the plant extract's compounds, such as saponin, anthraquinone, tannins, and flavonoids (Emamghoreishi et al., 2011).

The biological properties of PF are attributed to the high amount of coumarins present in the plant (Sadraei et al., 2013). Several studies have reported plant‐derived coumarins’ antioxidant and anti‐inflammatory effects (Fylaktakidou et al., 2004; Witaicenis et al., 2014; Borges Bubols et al., 2013). The antiinflammatory effects of ostiole, a natural coumarin, on histamine‐induced inflammatory responses were examined in peripheral blood mononuclear cells from children with ASD with/without allergies/asthma. The results showed that the ostiole exhibited selective enzymatic inhibitory activity of COX‐2 (Kordulewska et al., 2019). Additionally, the neuroprotective effects of coumarins have been demonstrated in a stroke model (He et al., 2012). Coumarin ostiole has been shown to improve cognitive function and the blood–brain barrier in a model of cerebral ischemia using the nuclear factor erythroid 2–related factor 2 pathway in the CA1 region of the hippocampus and antiapoptotic pathways (Chen et al., 2015). Taken together, the administration of PF ameliorated the behavioral symptoms of autism via its antiinflammatory and antiapoptotic properties.

This study has a limitation. ASDs in animal models are described by core ASD‐like behaviors (social communication deficits and repetitive behaviors) and some comorbid traits, including anxiety‐like behavior, motor deficits, and abnormal sensory processing. So far, despite the prevalence of comorbidities in autism patients, animal models have focused mainly on characterizing core behavioral traits. Maternal exposure to VPA not only produces deficits in core ASD‐like behaviors (social interaction and repetitive‐stereotyped behaviors) but also leads to one or more comorbid features, including altered sensory processing, anxiety‐like behavior, and disturbed motor functions. Accordingly, mainly due to this reason, we measured some comorbid traits in the present VPA‐induced animal model of ASD. Additionally, due to some technical issues, we did not measure core ASD‐like behaviors, which the readers should notice as the limitation of this work.

## 5 CONCLUSION

The present study demonstrates that prenatal injection of VPA on day 12.5 of gestation could induce autism‐like behavior in offspring. The increased neuronal death in the hippocampus's CA1, CA3, and DG subregions appears to be related to the elevated oxidative stress biomarkers and reduced antioxidant activity in the hippocampus. The alterations in Bax and Bcl‐2 confirmed the apoptosis of pyramidal neurons in the hippocampus of VPA‐induced animals. On the other hand, administration of PF extract could reverse ASD‐like behaviors through its antioxidant and antiapoptotic properties in pups. Future studies should investigate further the potential benefits and underlying mechanisms of PF extract.

## AUTHOR CONTRIBUTIONS

Conceptualization; writing; original draft preparation: all authors. Material preparation and data collection: Maryam Saadat, Hamid Reza Sameni, Abbas Ali Taherian, and Abbas Ali Vafaei. Writing—review and editing: Maryam Saadat, Hamid Reza Sameni, Abbas Ali Vafaei, and Payman Raise‐Abdullahi. All authors have read and agreed to the published version of the manuscript.

## CONFLICT OF INTEREST STATEMENT

The authors declare no conflicts of interest. No financial gain was made during the research.

### PEER REVIEW

The peer review history for this article is available at https://publons.com/publon/10.1002/brb3.3224.

## Data Availability

The data that support the findings of this study are available on request from the corresponding author. The data are not publicly available due to privacy or ethical restrictions.
